# Effects of transition metal carbide dispersoids on helium bubble formation in dispersion-strengthened tungsten

**DOI:** 10.1038/s41598-023-40421-0

**Published:** 2023-08-16

**Authors:** Ashrakat Saefan, Xingyu Liu, Eric Lang, Levko Higgins, Yongqiang Wang, Osman El-Atwani, Jean Paul Allain, Xing Wang

**Affiliations:** 1 Ken and Mary Alice Lindquist Department of Nuclear Engineering, Pennsylvania State University, University Park, PA 16802 USA; 2 Department of Materials Science and Engineering, Pennsylvania State University, University Park, PA 16802 USA; 3 Department of Nuclear, Plasma and Radiological Engineering, University of Illinois at Urbana-Champaign, Urbana, IL 61801 USA; 4 Department of Nuclear Engineering, University of New Mexico, Albuquerque, NM 87106 USA; 5 Materials Science and Technology Division, Los Alamos National Laboratory, Los Alamos, NM 87545 USA; 6 Energy and Environment Directorate, Pacific Northwest National Laboratory, Richland, WA 99354 USA

**Keywords:** Nuclear fusion and fission, Structural materials

## Abstract

The formation of helium bubbles and subsequent property degradation poses a significant challenge to tungsten as a plasma-facing material in future long-pulse plasma-burning fusion reactors. In this study, we investigated helium bubble formation in dispersion-strengthened tungsten doped with transition metal carbides, including TaC, ZrC, and TiC. Of the three dispersoids, TaC exhibited the highest resistance to helium bubble formation, possibly due to the low vacancy mobility in the Group VB metal carbide and oxide phases. Under identical irradiation conditions, large helium bubbles formed at grain boundaries in tungsten, while no bubbles were observed at the interfaces between the carbide dispersoid and tungsten matrix. Moreover, our results showed the interfaces could suppress helium bubble formation in the nearby tungsten matrix, suggesting that the interfaces are more effective in trapping helium as tiny clusters. Our research provided new insights into optimizing the microstructure of dispersion-strengthened tungsten alloys to enhance their performance.

## Introduction

Because of its excellent thermal properties, high sputtering resistance, and low tritium retention, tungsten (W) is the leading candidate material for plasma-facing components (PFC) in the divertor region of tokamaks like ITER. However, embrittlement of W at high temperatures due to recrystallization, at low temperatures due to ductile to brittle transition, and under irradiation due to defect formation are major concerns of W-based PFC for long-term operations^1,2^. It has been demonstrated that dispersing second-phase particles, such as transition metal carbides and oxides, in the W matrix is a promising approach to addressing the above limitations^3–5^. Dispersoids could pin grain boundaries and raise the recrystallization temperature. Annealing at elevated temperatures showed that grain growth in dispersion-strengthened tungsten (DSW) with 1.0 to 10 wt.% TiC, ZrC, and TaC particles was suppressed up to temperatures as high as 1800 °C, whereas rapid grain growth would occur in pure polycrystalline W at about 900 °C^6^. With high chemical affinity to oxygen, the carbide dispersoids attract oxygen impurities and clean the W grain boundaries of these impurities, thus effectively improving the coherence of grain boundaries and the ductility of W matrix^5^. Flexural tests found that DSW with 0.5 wt.% ZrC exhibited a much lower ductile-to-brittle-transition temperature (~ 100 °C) than pure W (~ 400 °C)^5^. Since there is a plethora of dispersoid-matrix interfaces and grain boundaries as defect sinks, it is believed that DSW will have a higher resistance to radiation-induced damage^7,8^.

The extreme environment in tokamak divertors pose many challenges for W-based PFC. The high-flux helium (He) ion irradiation along with the ensuing formation of bubbles and surface nanostructures is one of these critical issues^9^. Although the behavior of W under He irradiation has been intensively studied, only a few works focused on similar topics in DSW. Baldwin et al*.* compared the fuzz formation in multiple W alloys, including DSW with 1.5% TiC or 1 wt.% La_2_O_3_, exposed to D_2_-He plasma in the PISCEB-B simulator at 850 °C with 25–60 eV He ions to the ion fluence of 3.6 × 10^21^ ions/cm^2^. Results showed that all the DSW alloys and pure polycrystalline W developed 2–3 mm fuzz layers, suggesting that dispersoids may have a negligible impact on surface nano-structuring under the high He fluence^10^. However, different phenomena were also reported. Liu et al*.* found that the nano-structured layer formed on W doped with ZrC was obviously lower than the layer formed on pure W irradiated by 220 eV He ions to 1.0 × 10^22^ ions/cm^2^ at 900 °C. In addition, no surface morphology change could be observed on Y_2_O_3_ particles^11^. Similarly, Lang et al*.* studied the surface morphology of DSW with TiC, ZrC, and TaC exposed to 250 eV He up to 1.0 × 10^20^ ions/cm^2^ at 800 °C. Although dense tendrils were formed on TiC and nano-blisters were formed on ZrC particles, TaC surfaces did not exhibit any obvious damage in the scanning electron microscopy (SEM) images^12^. All these studies indicate that certain dispersoids may be more effective in suppressing the formation of He bubbles and surface nanostructures. It is worth noting that while research has demonstrated the surface fuzz formation is caused by the accumulation of He bubbles in W^13^, it remains unclear whether a similar mechanism is valid for the carbide dispersoids, so further research is needed to fully understand the correlation between bubble formation and surface nano-structuring in transition metal carbides.

Besides the dispersoid type, the role of W-dispersoid interfaces on He bubble formation is also less well-established. Many studies have demonstrated the preferred trapping of He at grain boundaries of W, as well as at the interfaces between dispersoids (e.g., carbides and oxides) and Fe matrix in dispersion-strengthened steels^14–17^. Therefore, large bubbles were formed right at these defect sinks. Unexpectedly, preferential bubble formation was not observed at the W-dispersoid interfaces in DSW with TiC or TaC exposed by ~ 30 eV He ions to 5 × 10^22^ ions/cm^2^ at 900–1100 °C^18^. It was suspected that the W-dispersoid interfaces could trap He as small clusters, which were invisible in the transmission electron microscopy (TEM) images. Similarly, in-situ irradiation in TEM was conducted on DSW with TiC using 1 MeV Kr ions at 800 °C, and nanosized voids were formed only near the TiC-W interface but not at the interface^8,19^. In order to design DSW with superior resistance to He irradiation, it is necessary to establish more thorough understandings of the effects of W-dispersoid interfaces on bubble nucleation and growth.

In this study, we applied He ion irradiation and TEM analysis to study the bubble formation in DSW materials doped with transition metal carbides, with a special focus on understanding the effects of carbide chemistry and W-dispersoid interfaces. As discussed above, most studies used He ions with kinetic energy less than 1 keV, as it is the energy range of He ions existing in the divertor regions of tokamaks. However, the low energy He would only penetrate a shallow layer (~ a few nm) in W specimens, posing two restrictions for us to achieve the research goals. First, the He bubbles generated by the low-energy He implantation are very close to the surface, which is a strong defect sink and could play a dominant role in controlling the defect kinetic. Therefore, the impact of dispersoid chemistry and the dispersoid-W interfaces on bubble formation would become negligible and difficult to analyze. Second, as the bubbles would only appear within a few nanometers below the surface, the region of interest for TEM analysis is quite small. To address these limitations, we used He ions with higher energies (200 keV and 2 MeV), which will penetrate deep into DSW samples. Moreover, as PFCs are also subjected to a high fluence of fusion-produced neutrons, which could penetrate deep into the material and produce He atoms via transmutation reactions^1^, irradiation studies using higher He energies would also provide more insights regarding bubble formation induced by neutron damage. By combining ex-situ TEM analysis for characterizing bubble distribution with in-situ heating in TEM for monitoring the dynamic process of bubble formation, we systematically compared the behavior of DSW doped with ZrC, TaC and TiC under He irradiation, revealing the fundamental roles of dispersoid chemistry and interfaces on bubble formation.

## Results

### Ex-situ TEM analysis of DSW irradiated by 200 keV He

**Figure 1:**
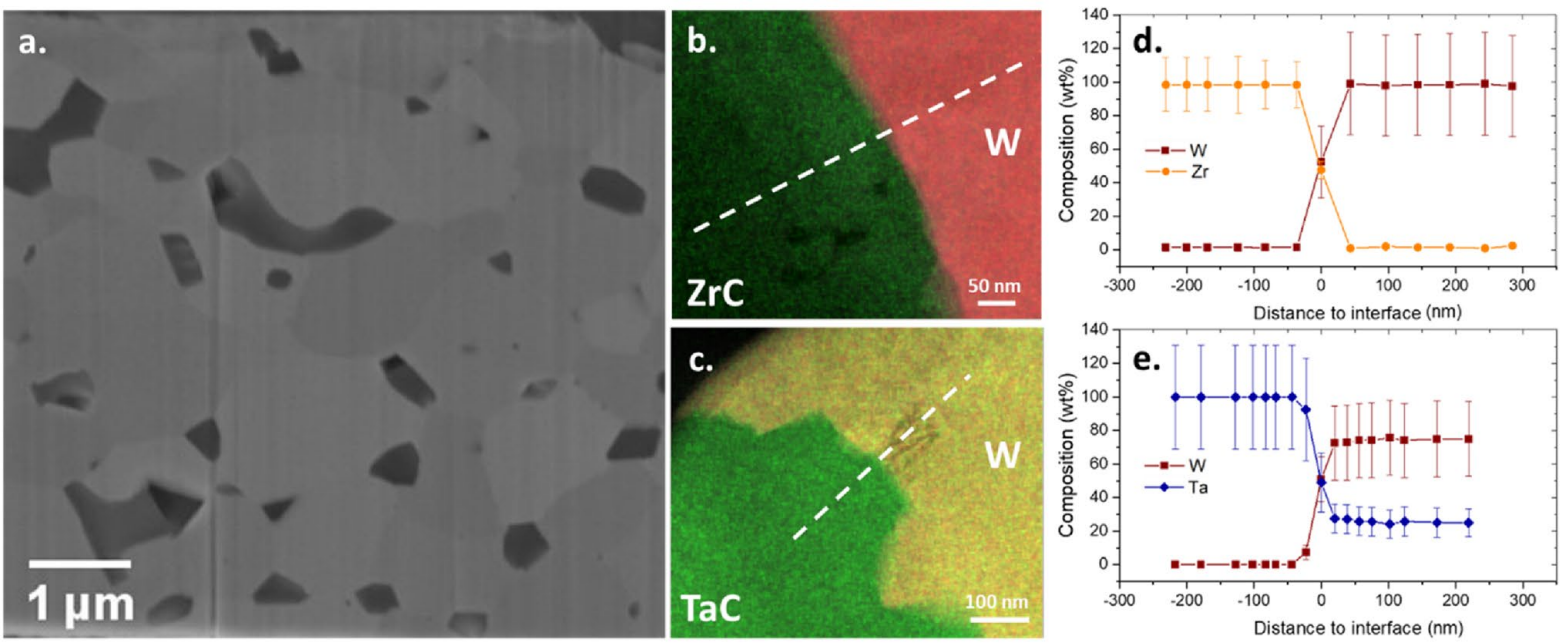
Microstructure of DSW. (**a**) SEM image of W–TiC; TEM-EDS element mapping of (**b**) W–ZrC and (**c**) W–TaC; 1D composition profile across the W-dispersoid interface in (**d**) W–ZrC and (**e**) W–TaC. The white dashed lines in (**b**) and (**c**) indicate the location for extracting 1D composition profile from the 2D element mapping. Error bars in (**d**) and (**e**) represent 3σ errors provided by the ESPRIT Spectrum software by assuming that the characteristic X-ray counts in EDS follow a t-distribution.

Figure 1a is an SEM image representing the typical microstructure of DSW materials fabricated by SPS. The carbide dispersoid particles appear as darker regions than the W matrix under the backscattered-electron imaging mode. The dispersoids are evenly distributed in the W matrix with most of them being intergranular. The average sizes of dispersoids and W grains are dependent on the type and concentration of the dispersoid particles. Detailed values have been reported in the Ref.^12^. Overall, the three DSW materials in this study contain dispersoid particles with an average size around 0.5–2 mm and W grains with an average size around 2–4 mm. More SEM images showing the dispersoid distribution are present in Supplementary Materials Section 1.

Because element intermixing near W-dispersoid interfaces may modify the nearby defect kinetics, we applied energy-dispersive X-ray spectroscopy (EDS) mapping to measure the element concentration profile across the interfaces. As shown in Fig. 1b–e, the intermixing of W and dispersoid metal elements is not greater than 50 nm near the interface in both W–ZrC and W–TaC, suggesting the impact of element intermixing on defect kinetics should be very localized. It is worth mentioning that our SEM–EDS analysis found that a high fraction of carbide dispersoids have transformed into oxides. The detailed SEM–EDS measurements are summarized in Supplementary Materials Section 1. Similar oxidation phenomena were also reported in other studies of DSW with transition metal carbides, probably due to the high chemical affinity of the carbides to oxygen^5,12,20^. To maintain consistency, we will continue using the term MC to refer to the dispersoids throughout the article, where ‘M' represents the transition metal element. However, it is important to note that the dispersoids are actually composed of both carbides and oxides. Since EDS is not the most sensitive method for measuring lighter elements like O and C, we only include the transition metal elements in our composition analysis in Fig. 1d,e. Atom probe tomography (APT) is being conducted now to obtain a more accurate measurement of C and O distribution near W-dispersoid interfaces, which will be reported in future publications. In addition, we noticed that there is a constant 20 wt.% Ta in the W matrix in W–TaC, even when it is more than 200 nm away from the interface (Fig. 1e). Note that the energies of characteristic X-rays from W and Ta are very close: the M peak is 1.709 eV for Ta and 1.774 eV for W, and the Lα peak is 8.145 eV for Ta and 8.396 eV for W. Therefore, the constant Ta concentration could be an artifact that resulted from the difficulty in separating the W and Ta peaks in the EDS spectrum. Again, APT analysis could help address this issue as APT distinguishes elements based on their atom mass numbers^21^. Nevertheless, our EDS results confirmed that the composition change near the W-dispersoid interface is restricted to a narrow region (less than 50 nm).

**Figure 2:**
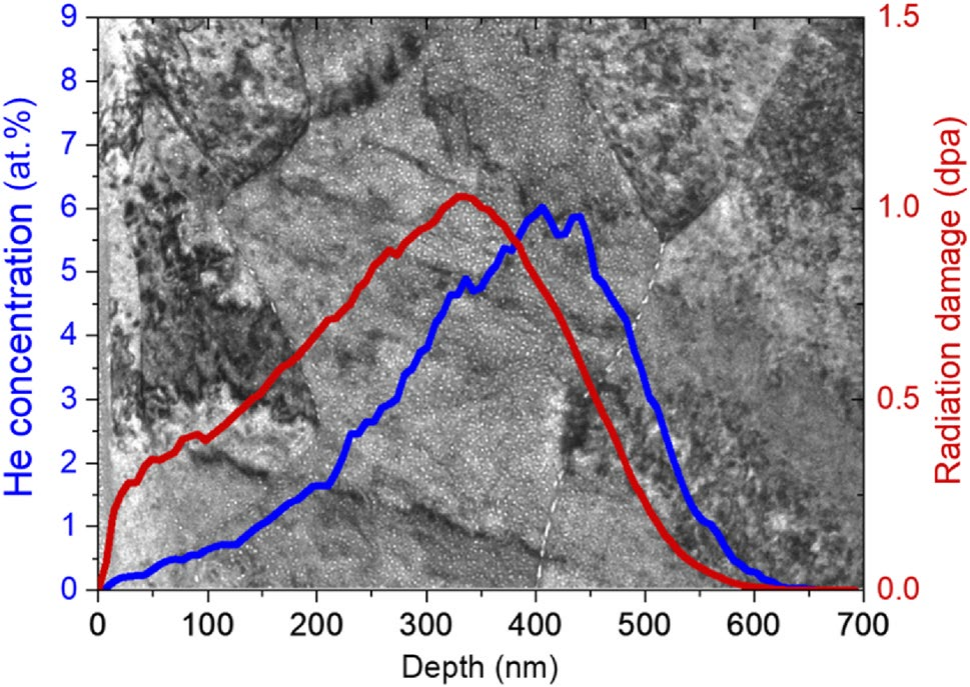
BF-TEM image showing distribution of He bubbles along damage profile in W matrix irradiated by 200 keV He^+^ ions. Red curve is radiation damage dose in dpa and blue curve is concentration of implanted He calculated by SRIM.

Figure 2 illustrates the distribution of He bubbles in the W matrix of W–TaC irradiated by 200 keV He ions, along with the resulting irradiation damage. The bright field (BF)-TEM image was acquired in under-focused condition, so bubbles appear as bright spots. The red curve represents the local radiation damage dose in displacements per atom (dpa), and the blue curve represents the concentration of implanted He in atomic percent (at.%). Both curves were calculated using the Stopping and Range of Ions in Matter (SRIM) code in the quick Kinchin–Pease mode with a displacement threshold energy of 90 eV and a mass density of 19.25 g/cm^3^ for W^22,23^. As shown in Fig. 2, high-density of He bubbles are located between 150 to 500 nm below the W surface, the depth of which has both high He concentration (≥ 1 at.%) and radiation damage (> 0.2 dpa) according to the SRIM calculation. Our following bubble analyses were all performed in this depth. It is also notable in Fig. 2 that large He bubbles are concentrated along grain boundaries, which will be discussed later in this section.

**Figure 3:**
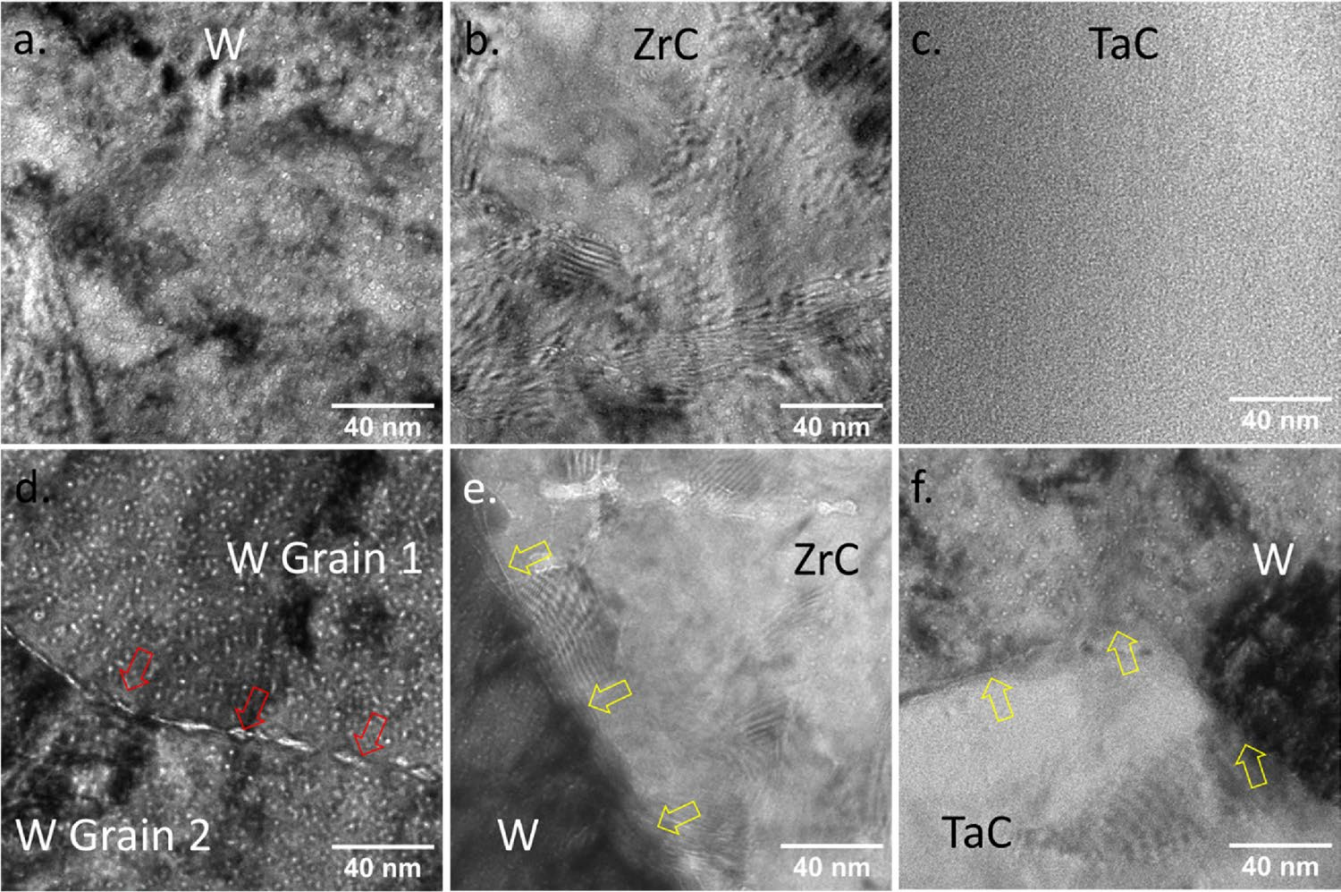
BF-TEM images showing bubble in (**a**) W matrix, (**b**,**c**) dispersoids, (**d**) near W grain boundaries, and (**e**,**f**) near W-dispersoid interfaces. (**a**–**c**) were acquired at 500 nm under-focus, (**d**) was acquired at 1.2 mm under-focus, and (**e**,**f**) were acquired at 700 nm under-focus, so bubbles would appear as bright spots in all the images. Red arrows in (**d**) indicate the locations of grain boundaries and yellow arrows in (**e**,**f**) indicate the location of W-dispersoid interfaces.

Additional BF-TEM analysis found that the distributions of bubbles in the W matrix of both W–ZrC and W–TaC samples are very similar. However, distinct differences were observed when we examined the bubbles in the dispersoids and near the interfaces between the W matrix and the dispersoid. Figure 3a–c compare the bubble formation inside the grains of W, ZrC, and TaC. Similar to W grains, nanometer-sized bubbles are formed in the ZrC dispersoid, although the average bubble size is slightly larger. In contrast, no bubble could be observed in the TaC dispersoid. Combining with in-situ TEM analyses presented in the next section showing that bubbles were formed in the TiC dispersoid, we conclude that TaC has the highest resistance to bubble formation among all three carbides. This trend is also consistent with the surface morphology changes discovered in a previous study: dense nanostructures (e.g., tendrils, pin holes, and blisters ) were developed on the surface of W matrix, TiC, and ZrC dispersoids, but no morphology change could be observed on the TaC surface when the DSW materials were implanted by 250 eV He to 1.0 × 10^20^ ions/cm^2^ at 800 °C^12^. Therefore, the surface morphology variation in DSW materials has been correlated to bubble formation in the He ion implantation region. It's noteworthy that the under-focused BF-TEM image featured in Fig. 3c exhibits several minuscule bright spots, which may appear akin to bubbles. However, we have verified that these microstructural features are not bubbles, as they do not transform into dark spots under over-focused imaging conditions. Additional details are furnished in Supplementary Materials Section 3.

The underlying mechanisms for the superior resistance of TaC dispersoids need future investigation. Nevertheless, the differences in defect kinetics, particularly vacancy mobility revealed by ab initio calculations may help shed light on understanding the unique property of TaC. Note that the dispersoids consist of both oxide and carbide phases. For the oxide phases, density functional theory (DFT) calculation found that the migration energies of oxygen vacancies in Ta_2_O_5_ (1.28–2.17 eV^24^) is obviously larger than that in ZrO_2_ (0.7–1.99 eV^25^) and TiO_2_ (0.9–2.1 eV^26^), indicating that the vacancy clustering by migration may be suppressed more effectively in Ta oxides and thus the growth of He bubbles was inhibited. For the carbide phases, DFT calculation also revealed that carbon vacancies in group VB carbides, e.g., TaC, has a larger migration barrier than that in group IVB carbides like TiC^27^. For example, the C vacancy migration energy is 4.00 eV in TaC and 3.79 eV in TiC. In addition, vacancy clusters containing one metal vacancy surrounded by six carbon vacancies has the lowest formation energy in group IVB carbides, whereas the vacancy clusters with the lowest formation energy in group VB carbides only contain metal vacancy surrounded by wo carbon vacancies^28^. Both factors will contribute to fewer and smaller vacancy clusters in TaC, and thus the suppressed bubble formation. Note that there are several other possible reasons that could also contribute to the lack of bubbles in TaC dispersoids, such as potentially a large vacancy formation energy in TaC, He trapping at small He-vacancy clusters which are non-visible in TEM images, and He trapping by other features. More systematic investigations are needed to fully uncover the underlying reason for the high resistance to bubble formation of TaC dispersoids.

**Figure 4:**
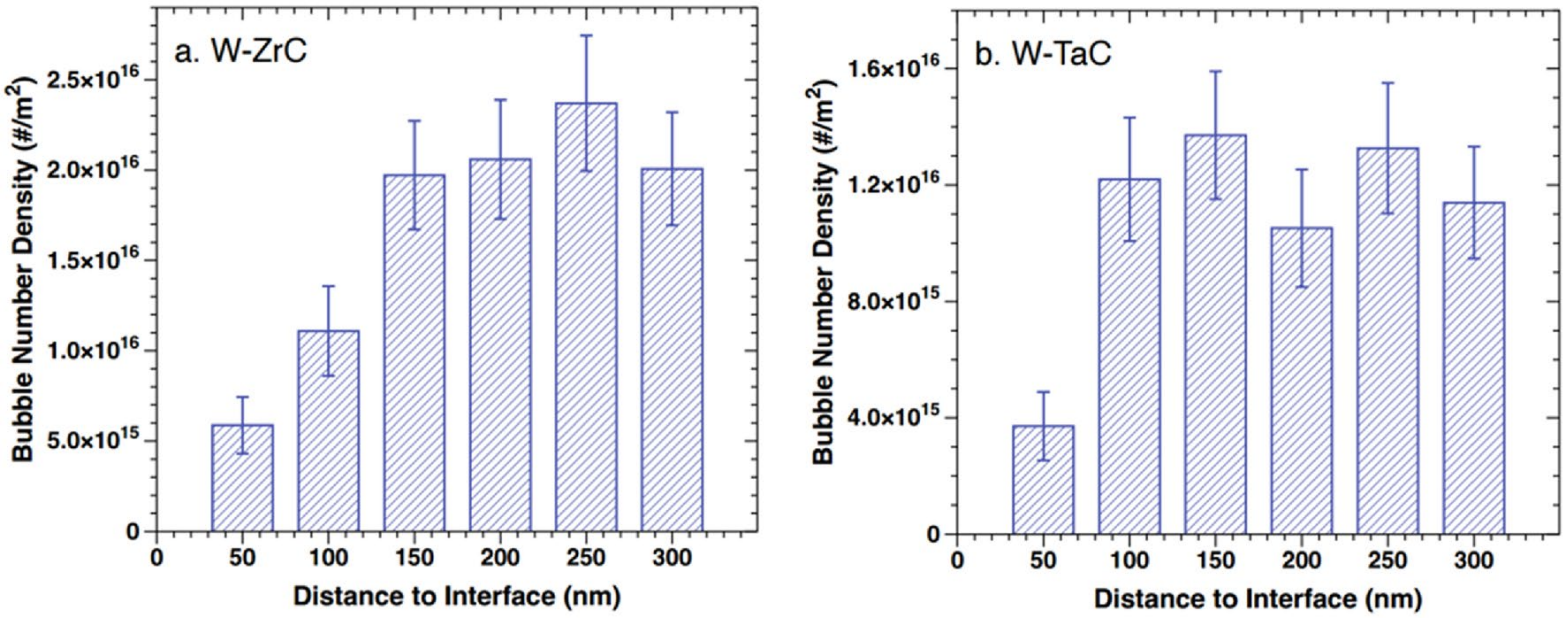
Local bubble number density in W as a function of distance to W-dispersoid interface in (**a**) W–ZrC and (**b**) W–TaC sample. The error bars were calculated by assuming the counts of bubbles in each distance follows a Poisson distribution.

Figure 3d,e compare He bubble formation near W grain boundaries and W-dispersoid interfaces at similar depths (~ 300–450 nm). Consistent with previous studies, grain boundaries serve as a strong defect sink for vacancies, He atoms, and He-vacancies complexes, resulting in large bubbles concentrated along the boundaries as marked by red arrows in Fig. 3d. Unexpectedly, no bubbles were observed at either W–ZrC or W–TaC interface marked by yellow arrows, which should behave similarly to grain boundaries as defect sinks and attract point defects. Moreover, careful examination of Fig. 3e,f found that bubble density near interfaces appears to be lower than within W grain interiors. To quantitatively verify the trend, we measured the local bubble number density as a function of distance to the W-dispersoid interfaces in both DSW materials. As depicted in Fig. 4, it is evident that the W-dispersoid interfaces suppress He bubble formation nearby, although the influence is highly localized: within about 50 nm near the interface, the bubble density is about 30% of that in the W grain interior, but at about 100–150 nm away from the interface, the local bubble density quickly returns to the same level as in the W bulk. As detailed in the next Section focusing on in-situ TEM analysis, similar behavior was also observed near the W–TiC interface, indicating that the suppressed bubble formation by W-dispersoid interface is a common phenomenon independent of the dispersoid type. Note that Fig. 4, the bubble area density in W–ZrC bulk (~ 2.0 × 10^16^/m^2^) appears to be higher than that in W–TaC bulk (~ 1.2 × 10^16^/m^2^), which is probably because the W–ZrC TEM sample is thicker than that of W–TaC.

**Figure 5:**
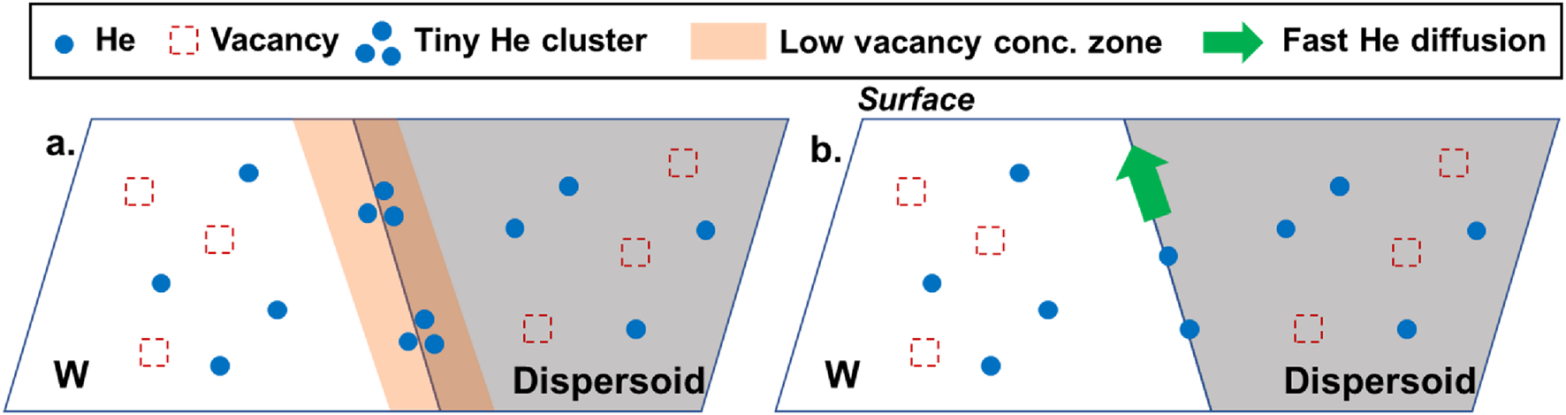
Schematics of two hypotheses for the lack of He bubbles at the W-dispersoid interface. (**a**) Hypothesis I: a low vacancy concentration zone exists near the interface that prevents tiny He clusters growing into large bubbles. (**b**) Hypothesis II: the interface acts as a fast diffusion channel for He atoms to escape from the interface to other sinks like surfaces.

We have proposed two hypotheses to explain the absence of bubbles at the W-dispersoid interfaces as depicted in Fig. 5. First, the vacancy concentration near the W-dispersoid interfaces may be too low to allow He clusters to grow into bubbles of a size detectable by TEM (Fig. 5a). Since the W-dispersoid interfaces are defect sinks, He atoms may be trapped there as tiny clusters (e.g., < 1 nm). However, several reasons may result in a scarcity of vacancies near the interface, thereby inhibiting bubble growth. The element intermixing at the interface, as shown in Fig. 1, could increase the vacancy migration barriers locally and reduce the vacancy flux getting to the interfaces. In addition, previous studies suggested that the misfit between the carbide dispersoids and the W matrix could effectively promote Frenkel pair recombination, thus efficiently absorbing excess vacancies near the interface^8,29^. It is also possible that due to the different atomic structures, the W-dispersoid interfaces offer larger free space than W grain boundaries, so more He could be trapped as tiny clusters and more vacancies are needed to form large He bubbles^30,31^. All these factors could contribute to insufficient vacancies at the dispersoid-W interface and suppressed formation of large bubbles. This hypothesis is similar to the theory put forth by El-Atwani et al. to explain the lack of large bubbles at W grain boundaries when the irradiation temperature is too low (< 800 °C) or the energy of He ions is too small (< 70 eV)^32,33^. Although the free volume of W grain boundaries can trap He^34^, if the irradiation temperature is not high enough, the mobility of vacancies and vacancy-He complexes in W matrix is insufficient, so they cannot reach grain boundaries to form large bubbles with the trapped He. Similarly, if the He ion energy is too small to cause atom displacement, no enough vacancies could be produced for the bubble formation at grain boundaries.

The second hypothesis is that the W-dispersoid interface may act as a fast diffusion channel, enabling He to migrate via the interfaces to other stronger sinks like W surfaces, and thus leaving few He atoms at the interfaces for large bubble formation (Fig. 5b). DFT calculation showed that the He migration energy along the W (100)-ZrC (100) interface is only 0.35 eV, significantly lower than that in the ZrC bulk (0.71 eV)^35^. This indicates that He is highly mobile along the W–ZrC interface at our irradiation temperature of 850 °C. A similar trend was also found in the W–TiC system^35^. The range of 200 keV He ions into W is approximately 350 nm, which is smaller than the average dispersoid sizes (~ 1 mm) in our DSW materials. Consequently, the implanted He could quickly migrate via the W-dispersoid interfaces that connect directly to the W surface, preventing large bubble formation at the W-dispersoid interfaces. This situation is different from He migration along grain boundaries. Molecular dynamics and statics calculations have found that depending on the migration direction, the migration energy of He along W grain boundaries can be as high as 0.8–1.3 eV, thus He will be trapped at the W grain boundaries^36^.

It is difficult to directly test these hypotheses since few microscopy techniques could image very small He clusters or track the He migration. Nevertheless, there is an indirect approach to examining the second hypothesis: we irradiated the DSW materials using He ions with a higher energy that penetrated deeper into the material, and then examined the bubble formation around dispersoids fully enclosed by the W matrix. In this case, He cannot escape from the W-dispersoid interfaces to the surface. If large He bubbles are still absent at the interfaces, it would disprove the second hypothesis and support the first hypothesis. The next section discusses this experiment.

### In-situ TEM analysis of DSW irradiated by 2 MeV He

**Figure 6:**
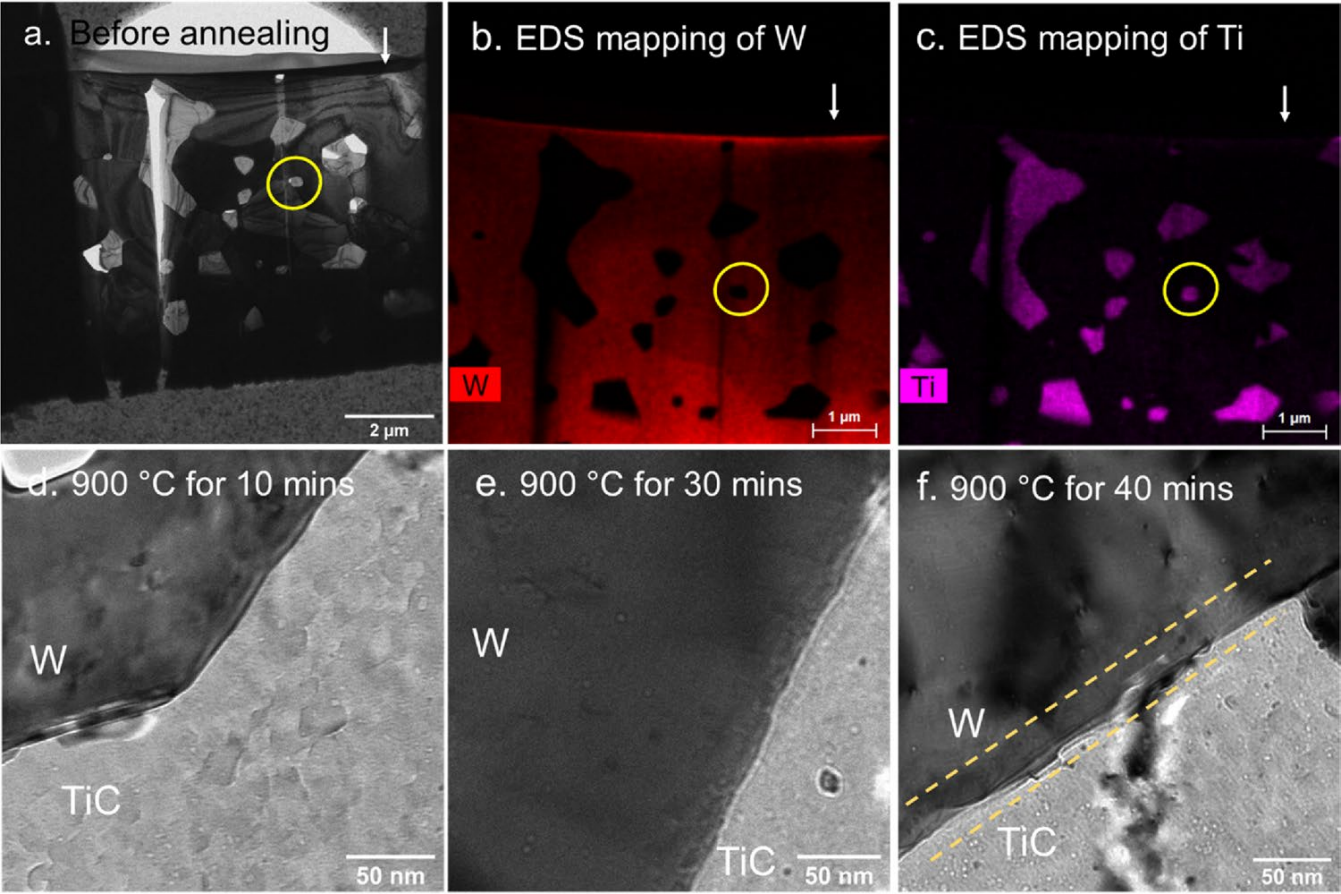
In-situ TEM analysis of W–TiC irradiated by 2 MeV. (**a**) Sample on the heating chip before annealing; (**b**) EDS mapping of W; (**c**) EDS mapping of Ti; (**d**) Under-focused BF TEM image when annealed at 900 °C for 10 min, (**e**) 30 min, and (**f**) 40 min. The white arrow in (**a**–**c**) indicates the position of irradiated surface, and the yellow circle indicates the dispersoid where zoomed-in images in (**d**,**e**) were acquired. The yellow dashed lines in (**f**) indicate the bubble denuded zone near the W–TiC interface.

Since the dispersoids in W–TiC have the smallest average size among all three DSW materials^12^, it would be the easiest to find a dispersoid particle fully enclosed by W matrix in W–TiC. Therefore, we started the high-energy He irradiation on W–TiC first. Specifically, a W–TiC bulk sample was irradiated by 2 MeV He ions to the fluence of 5 × 10^16^ ions/cm^2^ at room temperature. Based on SRIM calculation, the range of 2 MeV He ions in W is 2.7 mm and the He concentration at this depth is about 0.18 at.%. The irradiation temperature was chosen such that He migration along the W–TiC interfaces is highly limited, preventing it from escaping the material. At this temperature, vacancies in both W and TiC dispersoids are also immobile, so no He bubbles larger than 1 nm could be formed, which was demonstrated by our TEM analysis in the as-irradiated sample. To observe the dynamic process of bubble formation near the W-dispersoid interface, we performed an in-situ heating experiment in TEM. As shown in Fig. 6a, a thin foil of W–TiC prepared using the regular focused-ion beam (FIB) technique was transferred to the heating chip. Figure 6b,c are the element mapping based on EDS conducted before the annealing to confirm that multiple TiC dispersoids existed within the range of 2 MeV He ions. For the following microscopy analysis, we focused on the dispersoid which was about 2.2–2.5 mm below the surface and also fully enclosed by the W matrix.

During the in-situ heating experiment, the temperature of the thin foil was gradually raised from room temperature to 900 °C at a heating rate of 1 °C/s. The temperature was then held stable at 900 °C throughout the experiment, and we zoomed into regions near the W–TiC interfaces. As shown in Fig. 6d, nanosized bubbles started to appear in the TiC dispersoids after annealing for about 10 min. Bubbles in similar sizes also began to form inside the W grain when the sample was annealed for 30 min, and the bubble density in the dispersoid kept increasing with the annealing time. However, no bubble could be seen at the W–TiC interface. We continued the annealing for another 10 min and the trend remained the same. As shown in Fig. 6f, a bubble-denuded zone was developed near the interface after annealing for 40 min, which was about 30 nm wide on the W side and about 10 nm wide on the dispersoid side as denoted by yellow dashed lines. The absence of bubbles near the W–TiC interface is similar to the behavior of W–TaC and W–ZrC samples revealed by ex-situ TEM analyses, again supporting the conclusion that W-dispersoid interfaces suppress bubble formation. Moreover, since the TiC dispersoids studied here were fully enclosed by the W matrix, the absence of bubbles was unlikely to be caused by the fast migration of He via the interface to other stronger sinks such as the W surface. Therefore, the finding from the in-situ TEM disproved the second hypothesis and supported the first hypothesis, i.e., the W-dispersoid interfaces trap He as tiny clusters and insufficient vacancies exist at the interfaces for bubble growth. Considering that large He bubbles had been formed along grain boundaries under the same irradiation condition as shown in Fig. 3d, we can conclude that the W-dispersoid interfaces are more efficient than grain boundaries in retaining He as tiny clusters.

To reinforce our conclusions, we also subjected W–ZrC and W–TaC samples to the same 2 MeV He ion irradiation and conducted corresponding in-situ heating experiments in TEM. However, due to the larger dispersoid sizes, we were not able to locate any dispersoids that was fully enclosed by W and also within the range of 2 MeV He ions in the TEM specimens. Nevertheless, the in-situ heating experiments on W–ZrC and W–TaC still reveal trends consistent with those observed in the ex-situ TEM experiments, thereby further corroborating our conclusions. Specifically, we found nanosized bubbles in both the W matrix and ZrC dispersoids after heating, but did not observe any bubble formation in the TaC dispersoids, indicating that TaC has a higher resistance to He bubble formation than the other two dispersoids. Furthermore, no bubble was found at the W–ZrC interface, even after heating the samples to 900 °C for more than 40 min, suggesting the interface can help inhibit bubble formation. More details are presented in Supplementary Materials Section 3.

Note that the in-situ heating was conducted on a 100-nm thick foil as the TEM specimen, so the W–TiC interfaces shown in Fig. 6 were connected to the two surfaces of the thin foil. It is possible that the He trapped at the W-dispersoids may desorb from those surfaces during the annealing. However, this possibility is relatively low according to published thermal desorption spectroscopy (TDS) studies in polycrystalline tungsten. The desorption of He from W grain interiors usually occurs in the temperature of 400–1000 K and the desorption of trapped He at the grain boundaries occurs at about 1350 K^37,38^. In a TDS study of W doped with 1–10 wt% TiC, TaC, and ZrC, people found that He desorption was not completed even the samples were heated up to 1350 K^39^. Considering that the W-dispersoid interfaces can trap He to some extent, it is unlikely for the He to desorb completely from the interface during this in-situ TEM annealing experiment at 900 °C (~ 1173 K). It is also worth mentioning that although recent DFT studies indicated that the interfaces between W and different transition metal carbides have similar structures^35^, the He migration mechanisms in W–TiC may not be the same as in W–TaC or W–ZrC. Consequently, the observations drawn from the in-situ heating experiments of W–TiC may not unequivocally correspond to those from W–TaC or W–ZrC. More systematic studies on He desorption in the DSW materials are needed to conclusively rule out this possibility.

## Discussion and conclusion

The insights obtained from this work provide useful guidance for designing W-based materials for PFC with superior resistance to He bubble formation and the subsequent property degradation. First, since the bubble suppression is localized near the W-dispersoid interfaces and the size of dispersoids in our current DSW materials are relatively large (~ micrometers), the impact of dispersoids on the bubble formation in W matrix is found negligible. Nevertheless, refinement of the dispersoids to nanometer size by optimizing the spark plasma sintering techniques could substantially increase the dispersoid density and potentially help control the bubble formation in DSW materials. Second, the lack of He bubbles at the W-dispersoid interfaces presents a promising strategy for enhancing the mechanical properties of W-based PFCs under He implantation. Although nanocrystalline W has demonstrated improved mechanical properties than conventional polycrystalline W, previous research found that the accumulation of large bubbles and voids at grain boundaries would result in an obvious softening effect, substantially undermining the excellent mechanical properties of nanocrystalline W^40^. As bubble formation at the W-dispersoid interfaces is hindered, doping W with nanosized carbide dispersoids could potentially achieve similar improvements in mechanical properties to nanocrystalline W but without the detrimental softening effect under He implantation. Third, considering its higher resistance to bubble formation, doping TaC dispersoid with W would be a better choice than TiC and ZrC, assuming the impact on other PFC properties are similar. Finally, our studies indicated that the chemistry near the dispersoid-W interfaces could play an important role in modifying the local defect kinetics under He irradiation. Future studies are needed to thoroughly understand the distribution and chemical status of elements near the dispersoid-W interfaces and the impact on the properties of DSW.

It should be underscored that the He ion energies in this study (200 keV and 2 MeV) far exceeds the energies of He impinging upon the tokamak divertors (50–1000 eV). Consequently, readers should exercise caution when directly applying the results from this study to interpret the surface nanostructuring in DSW as PFCs in divertors. Nevertheless, this work brought in new understandings about the roles of dispersoid chemistry and W-dispersoid interfaces on the bubble formation in the bulk of W-based composite materials. Besides the exposure to the high fluence of He ions, W-based PFCs are also constantly bombarded by the 14.1 MeV fusion neutrons, which would produce significant displacement damage within the materials. Therefore, the high-energy He irradiation in this study offered a unique opportunity to investigate the coupled effects of He presence and displacement damage on bubble formation.

In summary, we investigated the effects of dispersoid chemistry and W-dispersoid interfaces on the He bubble formation in DSW materials. By comparing bubble formation in three different dispersoids of transition metal carbides (TaC, ZrC, and TiC), we concluded that TaC dispersoid has the highest resistance to bubble formation, probably owing to the low vacancy mobility in the Ta oxide and carbide phases. Unlike grain boundaries in W, the W-dispersoid interfaces did not exhibit preferred bubble growth. On the contrary, bubbles were absent along the interfaces and the bubble formation nearby was also suppressed, although the suppression effect only extended to tens of nanometers away from the interface. Based on in-situ TEM annealing experiments of DSW samples irradiated by 2 MeV He ions, we disproved the hypothesis that the fast migration of He along the W-dispersoid interfaces would lead to the suppressed bubble formation. Compared to W grain boundaries under the same irradiation condition, the absence of bubbles at the dispersoid-W interface indicates that the interface is a more efficient sink for sequestering He as tiny clusters. These findings from this research may offer valuable direction in the development of DSW materials for PFCs that exhibit enhanced resilience against helium bubble formation and the subsequent deterioration of their properties.

## Methods

DSW bulk samples doped with ZrC, TaC, or TiC dispersoid particles were fabricated using spark plasma sintering (SPS). Specifically, high-purity (> 99%) nanosized powders of W, TiC, ZrC, and TaC were carefully weighed and then mixed. The W,TiC, ZrC, and TaC powder size was about 500 nm, 80–130 nm, 20–40 nm, and 1000 nm, respectively. The mixture was ball milled for at least 12 h before consolidation in the SPS facility at 1800 °C under a uniaxial pressure and a DC current. More details of the fabrication and microstructures of DSW samples were discussed in the Refs.^12,41^. In this study, we used DSW materials containing 10 wt% ZrC or TaC dispersoids for the 200 keV He ion irradiation, and DSW containing 5 wt% ZrC, TaC, or TiC dispersoids for the 2 MeV He ion irradiation. For simplicity, we will refer to each DSW sample as W-MC, where ‘M' represents the metal element, without repeating the carbide concentration throughout the rest of the article. To prepare for the He ion irradiation, the fabricated bulk DSW samples were grounded and mechanically polished using standard metallographic techniques with the finishing step using 0.05 µm diamond suspension.

To generate different implantation depths, DSW samples were irradiated by either 200 keV He or 2 MeV He ions. The 200 keV He ion irradiation was conducted on W–ZrC and W–TaC samples using a 200 kV Danfysik Research Ion Implanter at the Ion Beam Materials Laboratory at Los Alamos National Laboratory. During the irradiation, the bulk samples were mounted on a Mo block using high temperature silver paste, and a thermocouple was attached to the copper block to monitor the sample temperature during irradiation. The 200 keV He ion implantation was conducted at 850 °C with a beam flux of approximately 2.0 × 10^13^ ions/cm^2^/s. The total irradiation fluence was 1 × 10^17^ ions/cm^2^. The 2 MeV ^4^He^+^ ion irradiation was performed on all three DSW materials (i.e., W–TiC, W–ZrC, and W–TaC) using the NEC Pelletron Accelerator at University of Illinois-Urbana Champaign. The samples were irradiated at room temperature to the fluence of 5 × 10^16^ ions/cm^2^ with a beam flux of approximately 1.4 × 10^12^ ions/cm^2^/s.

We used standard lift-out techniques with focused-ion beam (FIB) instruments to prepare thin foils of the implanted DSW samples for TEM analysis. To visualize He bubbles, we employed the through-focus technique in bright field (BF)-TEM images based on the Fresnel contrast mechanism, where bubbles appear as bright spots in under-focused condition and as dark spots in over-focused condition. Examples of BF-TEM images at different focus conditions are provided in Supplementary Materials 3. To measure the local bubble number density, we drew a spherical contour around each bubble and then used the Particle Analysis function in the ImageJ software to automatically count the bubble number. Energy-dispersive X-ray spectroscopy (EDS) mapping in both TEM and SEM was conducted to obtain the local compositions of DSW. For TEM-EDS, the scanning time was 5 min for each area to collect enough signals for composition measurement. The qualitative composition analysis was achieved by using the ESPRIT Spectrum (version 1.9) software from Bruker. For SEM–EDS analysis, the electron beam energy was 20 keV, and the element mapping was conducted using a frame of 1024 × 1024 pixels with pixel dwell time of 200 ms. The dead time was about 24% so the total time for one frame is about 2 min 25 s. The Aztec software (version 5.1) from Oxford Instruments was applied for the quantitative SEM–EDS analysis.

To observe the dynamic bubble formation process, in-situ heating in TEM was conducted on the W–TiC sample with pre-implanted He ions at room temperature. Specifically, a normal TEM lamella (~ 100 nm thick) was first prepared by FIB on copper grids and then was directly transferred to the SiN membrane on the Protochips heating chip. In-situ heating in TEM was achieved using the Protochips Fusion holder, which can instantly heat the lamellae up to 1200 °C while maintaining the maximum spatial resolution on the TEM instrument. In our experiments, both the heating and cooling rate were set as 1 °C/s and we heated some samples up to 900 °C. Note that although the direct FIB transferring can help us avoid most of the surface contaminations, the carbon re-deposition during the attachment of lamellae to the SiN membrane is still inevitable. To minimize the influence of carbon re-deposition, TEM analysis was only conducted in regions far away enough (at least 2 mm) from the carbon decomposition positions. All the microscopy analyses were conducted using instruments at the Materials Characterization Laboratory of Pennsylvania State University, including an FEI Talos F200X 200 keV TEM equipped with high-speed EDS for both in-situ and ex-situ TEM imaging, an FEI Helios 600i Dual Beam FIB/SEM for preparing thin foils, and an ThermoFisher Apreo 2 SEM for surface analysis.

### Supplementary Information

Supplementary Information.

## Data Availability

The datasets used and/or analysed during the current study are available from the corresponding author on reasonable request.
