# Supplementary material for: Effects of transition metal carbide dispersoids on helium bubble formation in dispersion-strengthened tungsten
Source: Sci Rep. 2023 Aug 16;13:13352. doi: 10.1038/s41598-023-40421-0 (PMC10432386; doi:10.1038/s41598-023-40421-0)
Supplement: Supplementary file 1 — Supplementary Information. [file 41598_2023_40421_MOESM1_ESM.docx]

**Supplementary Materials for**

**Effects of transition metal carbide dispersoids on helium bubble formation in dispersion-strengthened tungsten**

Ashrakat Saefan^1^, Xingyu Liu^2^, Eric Lang^3,4^, Levko Higgins^1^, Yongqiang Wang^5^, Osman El-Atwani^5^, Jean Paul Allain^1^, and Xing Wang^1, *^

1. Ken and Mary Alice Lindquist Department of Nuclear Engineering, Pennsylvania State University, University Park PA 16802, USA
2. Department of Materials Science and Engineering, Pennsylvania State University, University Park PA 16802, USA
3. Department of Nuclear, Plasma and Radiological Engineering, University of Illinois at Urbana-Champaign, Urbana IL 61801, USA
4. Department of Nuclear Engineering, University of New Mexico, Albuquerque, NM 87106, USA
5. Materials Science and Technology Division, Los Alamos National Laboratory, Los Alamos NM 87545, USA

***** Corresponding author: xvw5285@psu.edu

**1. Microscopy analysis of dispersion-strengthened tungsten materials using scanning electron microscopy**

As shown in **Fig. S1**, the distribution of dispersoid particles in the tungsten (W) matrix was characterized using scanning electron microscopy (SEM) and also energy-dispersive X-ray spectroscopy (EDS) in SEM. Based on the element mapping using SEM-EDS, we also measured the element concentrations in the dispersoid particles. The results are summarized in Table S1. In each DSW material, three different dispersoids were measured and the average concentrations are reported in the table, and the standard deviation of the three measurements is taken as the measurement uncertainty. It is clearly that in all three DSW materials, the dispersoids contain a high fraction of oxygen and the carbide dispersoids have been converted to a mixture of oxides and carbides. Note as EDS is not the most sensitive method for quantifying lighter elements like C and O, so the concentration values presented in Table S1 should be taken more for their qualitative insights rather than numerical precision.

**
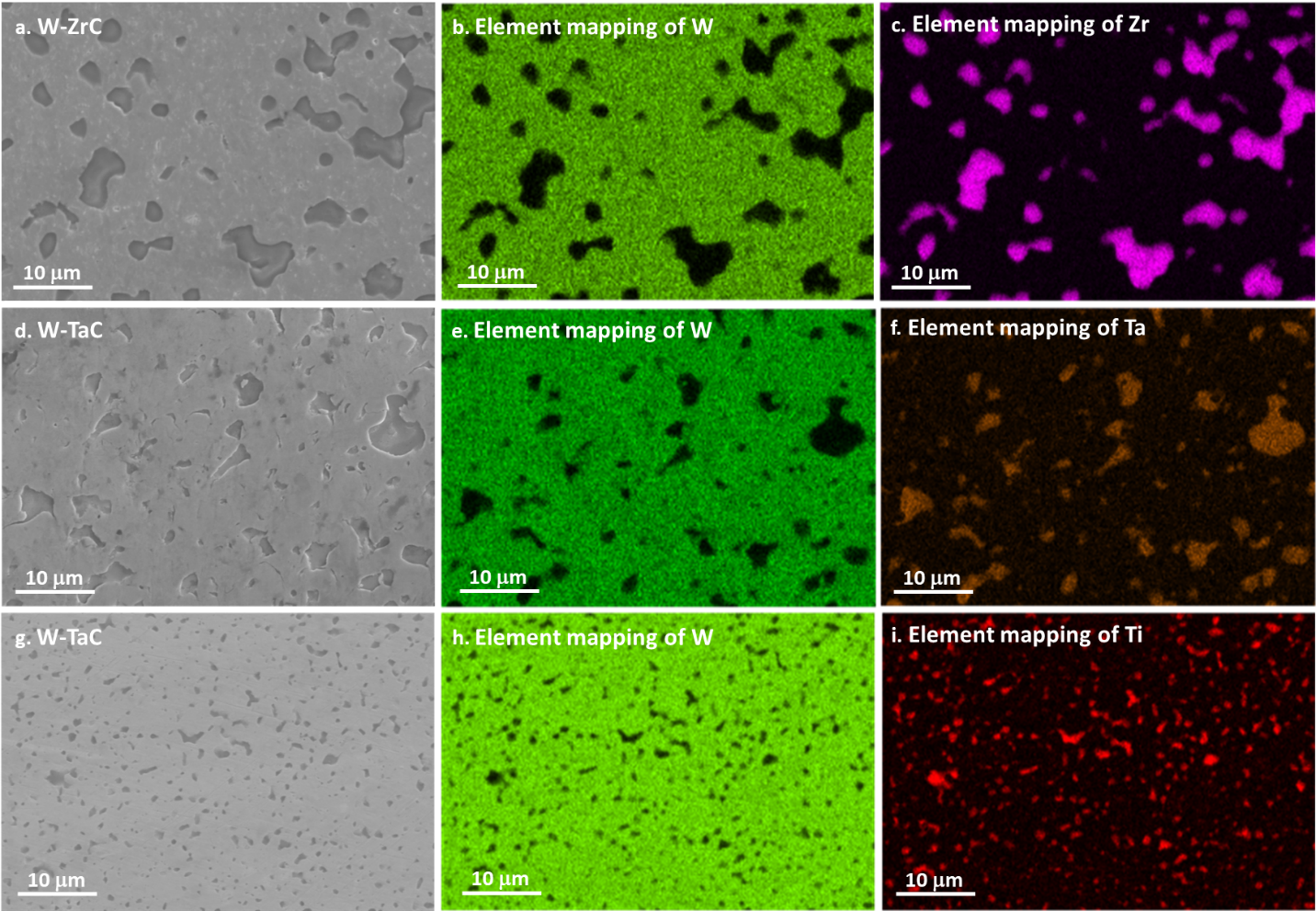
**

**Figure S1**. SEM image and SEM-EDS element mapping in (a-c) W-ZrC, (d-f) W-TaC, and (g-i) W-TiC.

**Table S1**. Element concentrations inside dispersoid particles based on SEM-EDS measurements.

| **Dispersoids in** | **Element concentration (wt%)** | | | |
| --- | --- | --- | --- | --- |
|  | **Transition metal** | **Oxygen** | **Carbon** | **Tungsten** |
| **W-ZrC** | 58.7±2.0 (Zr) | 25.1±1.3 | 7.8±0.5 | 8.3±3.8 |
| **W-TaC** | 73.2±2.7 (Ta) | 16.0±0.4 | 5.4±0.2 | 5.37±2.7 |
| **W-TiC** | 48.7±2.4 (Ti) | 9.3±1.5 | 5.40±0.3 | 36.2±3.6 |

**2. In-situ TEM heating of W-ZrC and W-TaC irradiated by 2 MeV He**

To further substantiate our conclusions, we subjected the W-ZrC and W-TaC bulk samples to the 2 MeV He ion irradiation at room temperature, then extracted thin foils from these samples using focused-ion beam (FIB) techniques. Following this, we proceeded to perform in-situ heating tests in transmission electron microscopes (TEM). We meticulously maintained identical experimental conditions to those used for the W-TiC samples to ensure a fair comparison.

The acquired bright field (BF)-TEM after heating the samples to 900 °C for 40 minutes are displayed in Fig. S2. Since the dispersoids in W-ZrC and W-TaC are relatively large, we cannot locate any dispersoids that were fully enveloped by the W matrix in these TEM specimens. Instead, extensive W-dispersoid interface was identified, as highlighted by the yellow arrows in Fig.S2(a) for W-ZrC and in Fig. S2(d) for W-TaC. Note that there is an apparent gap between the TaC dispersoid and the W matrix in Fig. S2(d). This gap was created immediately upon heating the TEM specimen up to 900°C. This likely results from differing thermal expansion coefficients between these two materials, thus rendering the W-TaC interface unfit for further TEM analysis. Nevertheless, the W-ZrC interface withstood the in-situ heating and zoomed-in TEM image in Fig. S2(b) clearly shows that large bubbles were formed in the ZrC dispersoids, but no bubble could be observed at the interface. Due to issues during the FIB sample preparation, the W material on the other side of the interface was considerably thicker than the ZrC side, making it challenging to determine whether bubbles formed in W using the same TEM image. By employing higher TEM magnification with a more under-focused condition, we confirmed the presence of bubbles within the W matrix, some of which are highlighted with red arrows in Fig. S2(c). Consequently, the in-situ heating experiment of W-ZrC once again demonstrates that the interface can assist in inhibiting bubble formation. For the W-TaC sample, we also observed a similar behavior to the ex-situ experiment. Specifically, nanosized bubbles were formed in the W matrix as shown in Fig.S2(f), but none could be detected in the TaC dispersoids in Fig. S2(e). Note that numerous black spots appear in this figure, which may be introduced by re-deposition during the FIB sample preparation. We confirmed that these black spots are not cavities (bubbles or voids) using the Fresnel contrast mechanism explained in the next section.

**
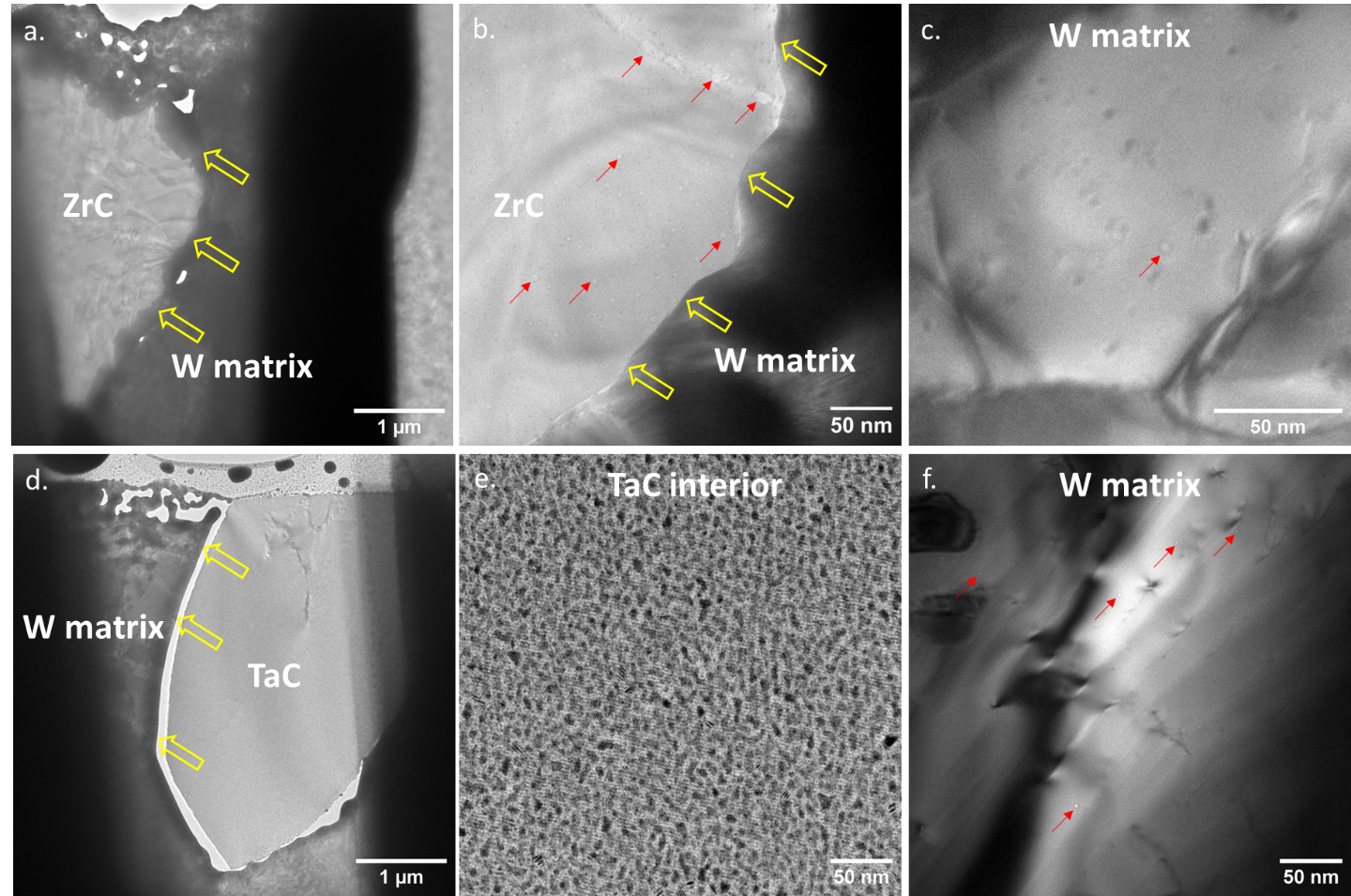
**

**Figure S2**. Under-focused BF-TEM images of W-ZrC and W-TaC samples after in-situ TEM heating at 900 °C for 40 minutes. (a) Overview image of W-ZrC specimen; (b) Zoomed-in image of W-ZrC interface; (c) Zoomed-in image of W matrix in W-ZrC; (d) Overview image of the W-TaC specimen; (b) Zoomed-in image of TaC dispersoid; (f) Zoomed-in image of W matrix in W-TaC. Yellow arrows in (a, b, d) indicate the interface locations, and red arrows in (b, c, f) highlight positions of some He bubbles formed after heating.

**3. Microscopy analysis of helium bubbles based on Fresnel contrast mechanism**

To confirm the microscale features observed in the bright field (BF)-transmission electron microscopy (TEM) images shown in Fig. 3 and Fig. 6 are bubbles, we applied the Fresnel contrast mechanism to every TEM image. The bubbles (or cavities) should have brighter intensities than the material matrix in under-focused conditions and lower intensities in over-focused conditions. **Fig. S3** below shows an example of the BF-TEM images acquired near the W-TaC interface at different focusing conditions, demonstrating that there are numerous bubbles in the W phase, but no bubble at the interface or the TaC dispersoid.


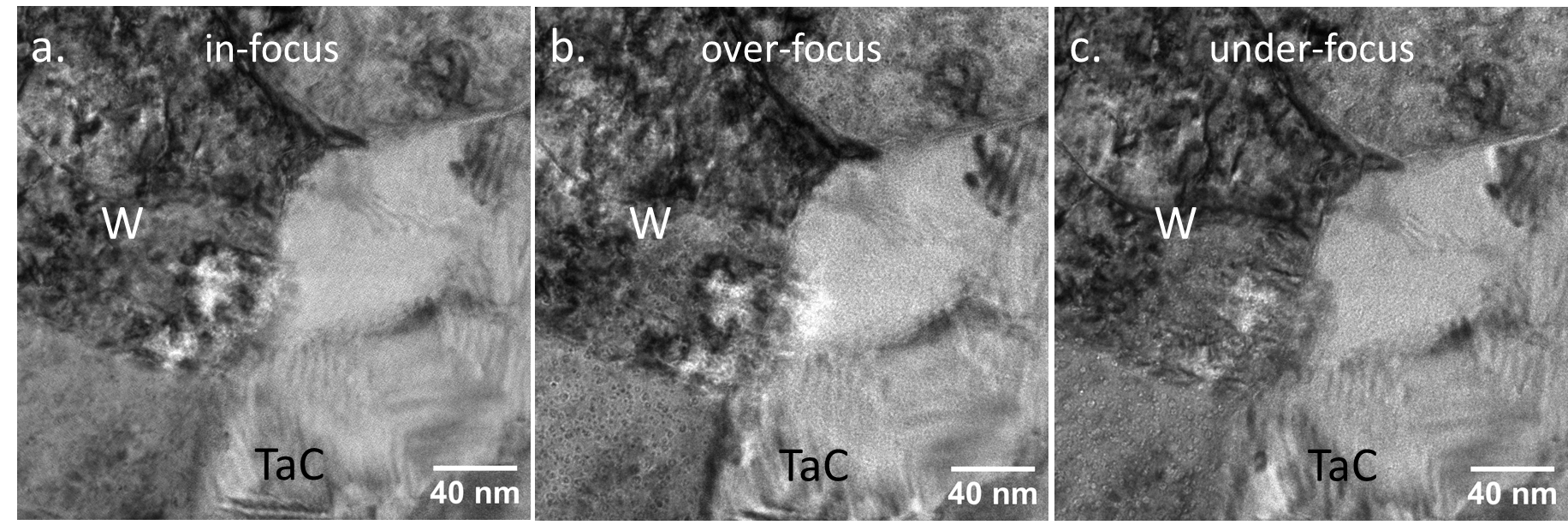


**Figure S3**. BF-TEM images of W-TaC interface at (a) in-focus condition; (b) 600 nm over focused condition; (c) -600 nm under focused condition.
